# Front-line treatment approaches in multiple myeloma: real-world data from a community-based oncology network

**DOI:** 10.3389/fonc.2026.1736892

**Published:** 2026-04-22

**Authors:** R. Steve Paulson, Santosh Gautam, Rohan Medhekar, Siraje Mahmud, Ayda Mirsalehi, Laura Ensley, Brian Shelley, Lorraine Brisbin, Monica Lisi, Vipin Khare, Shuchita Kaila, M. Yair Levy

**Affiliations:** 1 Texas Oncology Professional Association (PA), Dallas, TX, United States; 2 Johnson & Johnson, Horsham, PA, United States; 3 Precision Health Informatics, LLC, Dallas, TX, United States

**Keywords:** community oncology, daratumumab, frontline treatment, multiple myeloma, treatment patterns

## Abstract

**Background:**

The front-line (1L) treatment landscape for newly diagnosed multiple myeloma (NDMM) is rapidly evolving with guidelines endorsing triplet and quadruplet regimens as new standard-of-care. Despite this, real-world evidence on current treatment patterns for NDMM remain limited, especially in community settings, where most care is delivered. This study describes 1L treatment patterns for NDMM using recent data from a large US community oncology network.

**Methods:**

This retrospective, observational study analyzed structured and unstructured medical records of patients treated at Texas Oncology. Patients with NDMM (aged ≥18 years) who initiated 1L between January 2015 and December 2022 were included. Patient characteristics, 1L treatment patterns, and temporal trends were described.

**Results:**

3592 patients with NDMM who initiated 1L treatment were included. From 2015 to 2022, there was a nominal increase in the use of triplet regimens (59.9% to 87.6%). Quadruplet regimen use emerged in 2020 and rose from <1% to 9.4% in 2022. Daratumumab-based regimens numerically increased from 2% in 2019 to 29% in 2022. Daratumumab was integral to all quadruplet therapy regimens used by Texas Oncology centers and was predominantly utilized in the DVRd regimen.

**Conclusion:**

In this analysis, adoption of novel 1L regimens for NDMM, particularly daratumumab-based triplet and quadruplet regimens, was observed in recent years. However, the use of quadruplet regimens was still low in 2022, highlighting an opportunity for increased quadruplet uptake in the real-world. Future analysis will be essential to quantify the evolving uptake of quadruplet regimens in 1L and their impact on patient outcomes.

## 1 Introduction

Multiple myeloma (MM) is the second most common hematological cancer in the USA, with an estimated 36,000 new cases and 10,850 deaths in 2025 (1). The introduction of novel therapies in recent years has significantly prolonged progression-free survival (PFS) and enhanced survival rates in patients with MM (2). Despite these advances, MM remains incurable, with patients experiencing relapse and progressively poorer prognosis with each subsequent relapse (3). Several studies underscore the importance of optimizing front-line (1L) treatment strategies to potentially prolong long-term remission and enhance survival outcomes (4, 5).

The 1L treatment landscape for patients with newly diagnosed MM (NDMM) is rapidly evolving, with recent approvals of triplet and quadruplet regimens. These triplet and quadruplet regimens include drugs with different modes of action, which overcome drug resistance mechanisms (6). The first prospective studies to demonstrate the efficacy of triplet regimens used bortezomib (V)-lenalidomide (R)-dexamethasone (d; VRd) in NDMM (stem cell transplant [SCT] eligibility not stated), which showed an 18-month PFS rate of 75% (7), and Vd combined with cyclophosphamide for SCT-eligible NDMM, which reported an 88% response rate (8).

Subsequent studies into the use of quadruplet regimens for SCT-eligible NDMM included the 2020 phase 2 GRIFFIN trial that investigated the use of daratumumab (D) plus VRd (DVRd) compared to VRd (9). In this trial, 48-month PFS rate was 87.2% for patients treated with DVRd and 70.0% for patients treated with VRd (hazard ratio [HR]: 0.45, 95% confidence interval [CI]: 0.21-0.95; *P=*0.032) (9). In the 2024 phase 3 PERSEUS trial, the 48-month PFS rate was higher for patients treated with DVRd than patients treated with VRd (84.3% and 67.7%, respectively; HR: 0.42, 95% CI: 0.30-0.59; *P* < 0.0001) (10). Results of the PERSEUS trial led to Food & Drug Administration (FDA) approval of DVRd for SCT-eligible NDMM in July 2024 (11).

Triplet and quadruplet regimens have also shown efficacy in patients with SCT-ineligible NDMM. The 2017 phase 3 SWOG 077 demonstrated the efficacy of VRd compared to Rd (median PFS 43 months and 30 months, respectively; HR: 0.71, 95% CI: 0.56-0.91; *P* = 0.0018) (12). Similarly, in the 2019 phase 3 MAIA study, median PFS was significantly longer in patients with SCT-ineligible NDMM treated with DRd compared to those receiving Rd (61.9 versus 34.4 months; HR: 0.55, 95% CI: 0.45-0.67; *P* < 0.0001) (13). Estimated 60-month overall survival (OS) rates were 66.6% and 53.6% in the DRd and Rd groups, respectively (median OS not reached versus 65.5 months; HR: 0.66, 95% CI: 0.53-0.83; *P* = 0.0003) (13). Results of the MAIA trial led to the approval of DRd for the treatment of SCT-ineligible NDMM by the FDA in the USA in June 2019 (14). Quadruplet regimens have also been investigated for the treatment of SCT-ineligible NDMM. In the 2024 phase 3 IMROZ trial, patients with SCT-ineligible NDMM treated with isatuximab plus VRd had longer 60-month PFS rates than patients treated with VRd (63.2% versus 45.2%; HR: 0.60, 98.5% CI: 0.41-0.88; *P* < 0.001) (15). Isatuximab plus VRd was approved by the FDA for treatment of SCT-ineligible NDMM in September 2024 (16). More recently, in the 2025 phase 3 CEPHEUS trial, another quadruplet regimen, DVRd, was shown to improve OS (HR: 0.85, 95% CI: 0.58-1.24) and PFS (HR: 0.57, 95% CI: 0.41–0.79; *P* = 0.0005) compared to VRd in SCT-ineligible patients with NDMM (17). DVRd for SCT-ineligible patients with NDMM was granted FDA approval in January 2026 (18).

These novel 1L triplet and quadruplet regimens have the potential to provide benefits to patients with NDMM, as demonstrated in their respective clinical trials. Consequently, triplet and quadruplet regimens are recommended in National Comprehensive Cancer Network^®^ (NCCN^®^) Clinical Practice Guidelines in Oncology (NCCN Guidelines^®^) for treatment of NDMM, graded at the highest level of evidence (19). However, clinical trial data are not always representative of the general population and data on the real-world uptake of novel 1L treatment regimens, particularly in community settings, remain limited. The role of community clinics in providing care for patients with MM is crucial, as most patients with MM in the USA will receive treatment within these settings (20). Therefore, the aim of this study was to conduct a descriptive analysis of 1L treatment patterns for NDMM using data from one of the largest community oncology practice networks in the USA.

## 2 Methods

### 2.1 Study design

This retrospective, observational study analyzed structured and unstructured medical records of patients with NDMM treated at Texas Oncology, one of the largest community practice networks in the USA, with over 500 oncologists across more than 180 centers.

### 2.2 Patients

All patients at participating centers were included in this study if they met the following inclusion criteria:

- Had a first MM diagnosis (ICD-10-CM C90.0x) recorded in the iKnowMed electronic medical record database between January 1, 2015, and December 31, 2022.
- Initiated treatment with NCCN recommended MM drugs on or after the first MM diagnosis date, identified by chart review.
- ≥18 years old at the time of treatment initiation.

Patients were excluded from the study if they had:

- A diagnosis of non-MM cancer excluding plasmacytomas or basal/squamous cell carcinoma during the baseline period (defined below).
- Evidence of amyloid light-chain amyloidosis at any time during the study period.
- A record of SCT or clinical trial enrollment before 1L initiation.

### 2.3 Data collection

This study utilized a suite of primary databases, including the electronic medical record database iKnowMed and the molecular databases COPIA and CoPath. These databases include patients treated by Texas Oncology’s community oncology practice, which treats over 90,000 new cancer patients annually. For primary data collection, the investigators extracted and verified the accuracy of subject data. For data not obtained from a primary source, such as claims and electronic health records, investigators reviewed the data quality and relevance to meet the minimum requirements for all study objectives. The network has its own decision pathway tool, Clear Value Plus, which leveraged clinical data from iKnowMed to assess appropriate treatment recommendations, including line of treatment determination, using NCCN guidelines.

### 2.4 Assessments and outcomes

The index date was defined as the initiation date of 1L therapy. The baseline period was established as the six months preceding the index date. The follow-up period started at the index date and continued until the occurrence of one of the following events: death, initiation of hospice care, lapse of 60 days since last clinic visit, or the data cut-off date. Baseline patient characteristics (age, sex/gender, ethnicity, and race), clinical characteristics (International Staging System [ISS] staging and Eastern Cooperative Oncology Group performance status [ECOG PS]), primary insurance type, and calendar year of the start of 1L treatment were extracted from electronic medical records. 1L treatment regimens initiated between 2015 and 2022 were identified and stratified as monotherapy, doublet, triplet, and quadruplet therapies. Information on the types of daratumumab-based 1L treatment regimens initiated between 2019 and 2022 was also collected. Furthermore, bortezomib- and daratumumab-based 1L treatment regimens were stratified by patient age.

### 2.5 Ethics

Patient consent was not required for this study. This study was exempt from review by an IRB as the iKnowMed electronic medical record database is compliant with the Health Insurance Portability and Accountability Act (HIPAA), does not involve interaction or interview with any patients and does not include any individually identifiable data. The study was also exempt from IRB review as it was retrospective and observational. Personal patient data was not extracted or processed and study reports contained aggregate data only, without identifying individual patients.

### 2.6 Statistical analysis

Sample size calculations were not performed due to the descriptive nature of this study. All patients that met the study eligibility criteria were included in the analysis. Descriptive statistics were used. For age stratification, treatments were grouped into daratumumab-based and bortezomib-based regimen groups. For continuous measures, mean, standard deviation, and median are reported, and for categorical measures, the number and percentage of patients are reported. Investigators had full access to the database population. Missing data were not imputed and were reported as unknown where applicable.

## 3 Results

Overall, 10,520 patients with a diagnosis of NDMM between January 1, 2015, and December 31, 2022, were assessed for eligibility (**Supplementary Figure 1**). A total of 3592 patients with NDMM who initiated 1L treatment met the inclusion criteria and were included in this analysis (**Table 1**). The mean age at diagnosis was 68.4 years (standard deviation: 11.3) and most patients were male (55.0%) and white (68.5%). Most patients (67.6%) had an ECOG PS of 0–1 and the most common ISS disease stage in this patient population was Stage III (33.8%).

**Table 1:** Patient demographics and clinical characteristics.

Characteristics	Patients receiving 1L treatment (N = 3592)
Mean (SD) age at new diagnosis, years	68.4 (11.3)
Female, n (%)	1616 (45.0)
Race, n (%)	
American Indian or Alaska Native	8 (0.2)
Asia	71 (2.0)
Black or African American	490 (13.6)
Other and Unknown	564 (15.7)
White	2459 (68.5)
Ethnicity, n (%)	
Hispanic or Latino	681 (19.0)
Not Hispanic or Latino	2719 (75.7)
Unknown	192 (5.3)
Primary insurance type, n (%)	
Commercial	2235 (62.2)
Medicare/Medicaid	1346 (37.5)
Unknown	11 (0.3)
ISS staging, n (%)	
Stage I	628 (17.5)
Stage II	990 (27.6)
Stage III	1215 (33.8)
Unknown	759 (21.1)
ECOG Performance Status, n (%)	
0	1323 (36.8)
1	1105 (30.8)
≥2	170 (4.7)
Unknown	994 (27.7)
Calendar year of start of 1L, n (%)	
2015	344 (9.6)
2016	409 (11.4)
2017	410 (11.4)
2018	494 (13.8)
2019	440 (12.3)
2020	353 (9.8)
2021	546 (15.2)
2022	596 (16.6)
Median follow-up duration, months	23.8

1L, front-line; ECOG; Eastern Cooperative Oncology Group; ISS, International Staging System; SD, standard deviation.

### 3.1 1L treatment regimen distribution

**Figure 1:**
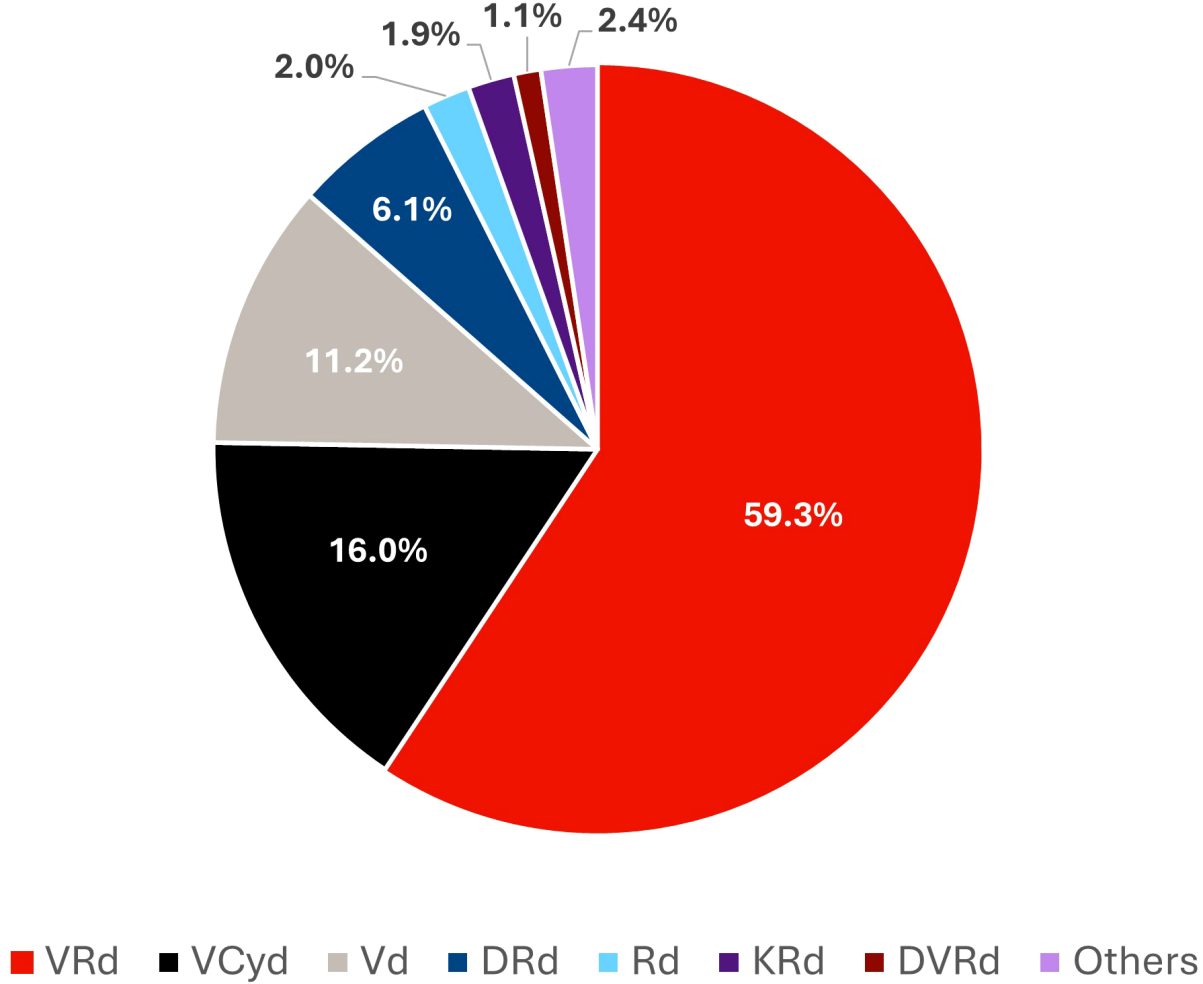
Distribution of 1L treatment regimens in the study population. 1L, front-line; DRd, daratumumab-lenalidomide-dexamethasone; DVRd, daratumumab-bortezomib-lenalidomide-dexamethasone; KRd, carfilzomib-lenalidomide-dexamethasone; Rd, lenalidomide-dexamethasone; VCyd, bortezomib-cyclophosphamide-dexamethasone; Vd, bortezomib-dexamethasone; VRd, bortezomib-lenalidomide-dexamethasone.

The most frequent 1L treatment regimens were VRd (59.3%), V-cyclophosphamide-d (VCyd; 16.0%), Vd (11.2%), DRd (6.1%) and Rd (2.0%; **Figure 1**). A stratified analysis by patient age revealed distinct patterns of use across treatment regimens. A numerically higher proportion of patients receiving daratumumab-based regimens were ≥75 years old than patients receiving bortezomib-based regimens (53.3% versus 46.7%; **Supplementary Figure 2**).

### 3.2 1L treatment trends

**Figure 2:**
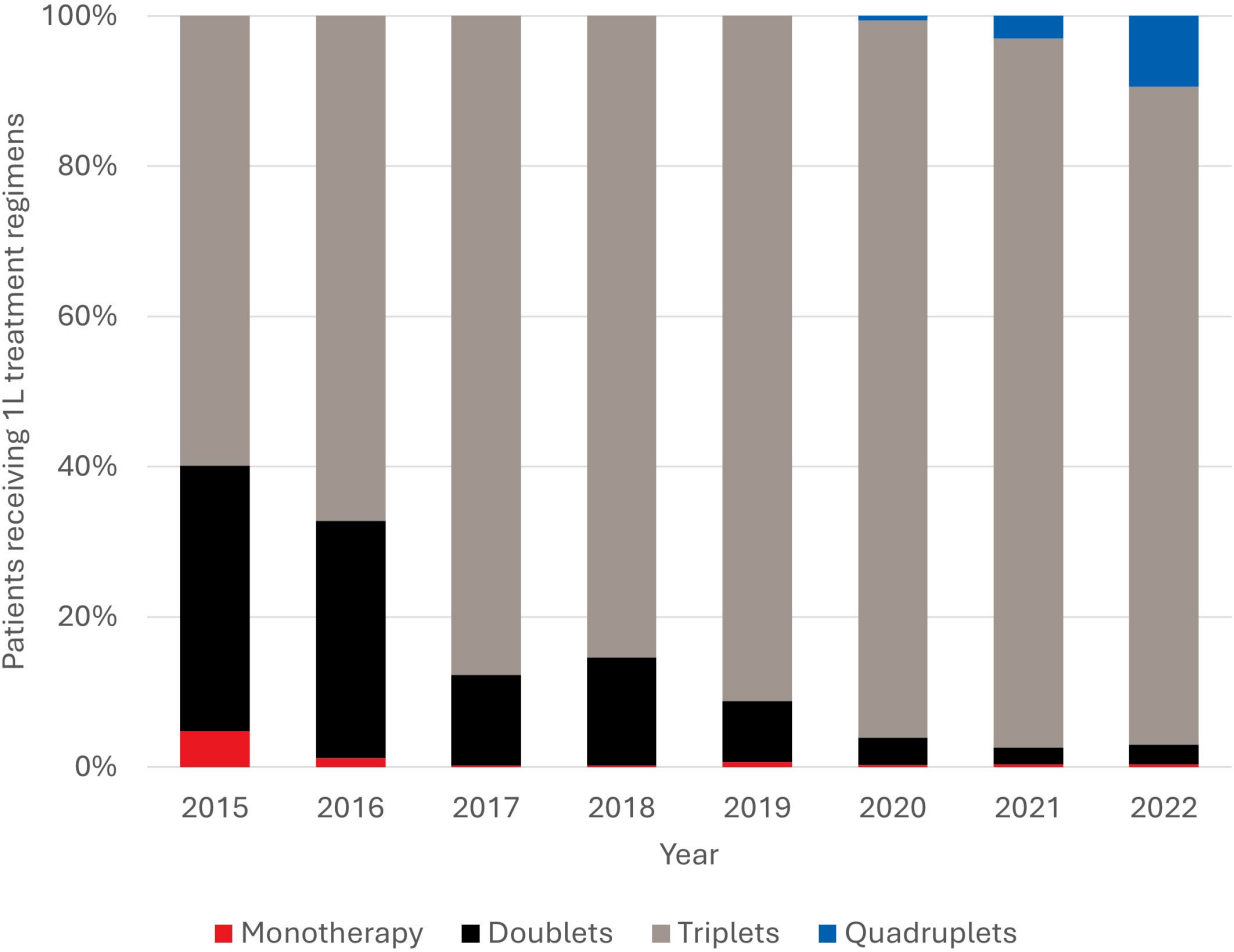
Trends in use of 1L treatment regimens between 2015 and 2022. 1L, front-line.

From 2015 to 2022, there was a nominal decline in the use of monotherapy (4.7% to 0.4%) and doublet regimens (35.4% to 2.7%; **Figure 2**). During the same period, triplets became increasingly utilized (59.9% to 87.6%; **Figure 2**). Quadruplet regimens emerged from 2020 onwards, rising from less than 1% to 9.4% by 2022. Among the 71 patients receiving quadruplet regimens, all were daratumumab-based with DVRd (n=41, 57.7%) and D-carfilzomib (K)-Rd (DKRd; n=26, 36.6%) being the most predominant regimens.

### 3.3 Daratumumab-based regimens

**Figure 3:**
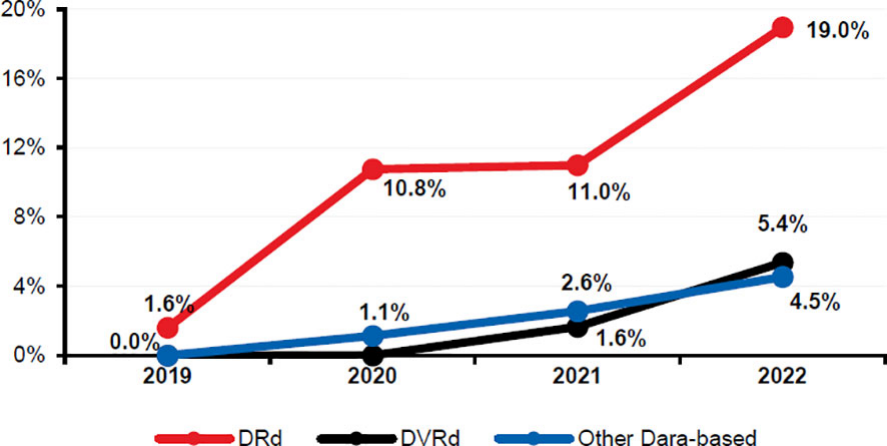
Trends in the use of daratumumab-based 1L regimens between 2019 and 2022. Other dara-based regimens include any treatment regimen which included dara, excluding DRd and DVRd. 1L, front-line; Dara, daratumumab; DRd, daratumumab-lenalidomide-dexamethasone; DVRd, daratumumab-bortezomib-lenalidomide-dexamethasone.

The use of daratumumab-based regimens numerically increased from 2% in 2019 to 29% in 2022, and DRd was the most frequently used daratumumab-based 1L regimen (**Figure 3**), coinciding with the approval of these regimens by the FDA. DVRd use started in 2021 at 1.6% and numerically increased to 5.4% in 2022 (**Figure 3**).

## 4 Discussion

This descriptive analysis of 1L real-world treatment patterns for patients with NDMM reveals important insights into current practices observed in a community setting. These findings showed that most patients were treated with triplet regimens, with VRd being the most frequently used regimen. This trend was accompanied by a steep drop in the use of 1L doublet regimens and an almost complete cessation of monotherapy use. Notably, there has been a marked numeric increase in the use of novel and more efficacious daratumumab-based regimens, rising from 2% in 2019 to 29% in 2022.

As most patients with MM will receive treatment in community settings (20), it is important to ensure that patients treated within community practices receive novel and efficacious treatment. However, it has been shown that physicians working within community settings are later adopters of novel treatment approaches and have been historically more likely to prescribe monotherapy or doublet treatment regimens than physicians working within academic centers (21). The community practice network analyzed in this study are increasingly adopting novel quadruplet regimens, which is consistent with a previous study of another large community oncology practice network in the USA (increase from 1.9% in 2018 to 39.1% in 2022) (22). Similarly, the decrease in the use of doublet regimens that was observed in this study, was also observed in the previous study of a large community oncology practice network (decrease from 25.8% in 2018 to 8.7% in 2022) (22). Nevertheless, the use of these regimens is not at the optimal level and represents an opportunity for further adoption of novel therapies.

Results of the phase 3 MAIA study showed that DRd improved survival outcomes of SCT-ineligible patients with NDMM compared to Rd (13). These results led to FDA approval of this regimen in 2019 (14), which may explain the increased use of DRd in this analysis from 2019 onwards. Although DRd and VRd have not yet been compared head-to-head in a clinical trial setting, an indirect treatment comparison of two clinical trials (S0777 and MAIA) showed that SCT-ineligible patients with NDMM had a greater PFS benefit with DRd than with VRd (23). The efficacy of DRd for the treatment of SCT-ineligible patients with NDMM has also been demonstrated in real-world studies. In the TAURUS chart review study, patients treated with DRd had a lower risk of disease progression and death compared to patients treated with VRd in the real-world (HR: 0.35, 95% CI: 0.17-0.73) (24). Similarly, the PEGASUS study demonstrated that patients treated with DRd in the MAIA trial had significantly lower risk of disease progression compared to patients receiving Rd or VRd treated in community-based oncology practices in the USA (25). These published real-world data illustrate the potential benefits of increased use of DRd in clinical practice.

While the adoption of quadruplet regimens started to increase from 2020 in this study, the overall use of quadruplets in this community practice network was still low (9.4%) in 2022, highlighting an opportunity for improvement in quadruplet treatment uptake in the real-world. The efficacy of quadruplet regimens has been demonstrated across multiple clinical trials. The IMROZ trial demonstrated the increased efficacy of isatuximab-VRd compared to VRd for 1L treatment of NDMM (15). The phase 2 GRIFFIN trial showed that the addition of daratumumab to VRd improved complete response rate and minimal residual disease negativity in SCT-eligible patients with NDMM, compared to VRd (9). Results from GRIFFIN may have contributed to the increased utilization of DVRd observed in this analysis between 2020 and 2022. Subsequently, DVRd was approved by the FDA for the treatment of SCT-eligible and SCT-ineligible patients with NDMM based on the results of the phase 3 PERSEUS and CEPHEUS trials, respectively (10, 11, 17, 18). These data, together with the recent FDA approval of DVRd for SCT-eligible and SCT-ineligible patients with NDMM (11, 18), have established quadruplet regimens as an effective treatment option in the 1L. This is further exemplified by the recommendation of 1L quadruplet regimens in NCCN guidelines. This change in the treatment landscape is expected to increase the adoption of quadruplet regimens in the coming years, as there is often a delay between clinical trial data publication and adoption of these treatments into community settings (26).

Triplet and quadruplet regimens are recommended in NCCN guidelines for treatment of NDMM, graded at the highest level of evidence (19). Results of systematic literature reviews, meta-analyses and consensus guidelines also show a strong recommendation for the use of triplet and quadruplet regimens in the 1L (27–29). However, the drivers that impact treatment selection by physicians in the real-world are not solely reliant on treatment guidelines, and physicians may select therapies outside these recommendations for practical reasons. For example, a previous study found that ‘health insurance covering full cost of treatment’ was one of the most common physician-reported reasons for treatment selection in MM across all lines of treatment (30). Insurance coverage may have impacted the treatment patterns observed in this study, as most (62%) patients had commercial insurance which could have enabled a broader availability of treatment. Further study is needed to assess the drivers of treatment selection in the real-world for 1L treatment of patients with NDMM, through the use of physician interviews and surveys. Such studies will measure changes in 1L quadruplet regimen use and gather changes in physician opinion on quadruplet regimens.

Limitations of studies utilizing real-world clinical data include the potential for miscoding and errors, and structured data fields often not fully capturing detailed clinical characteristics, which limits our understanding of the patient population. Potential sources of bias in observational studies include selection bias, misclassification, and confounding, and could represent limitations of this descriptive analysis. The findings of this study are based on data exclusively from one community oncology practice network and included patients who received at least one NCCN-recommended MM treatment and excluded those with amyloid light-chain amyloidosis to avoid inclusion of patients receiving atypical treatments, meaning that the patterns of care and patient demographics may not be generalizable to other community practices or clinical settings. No subgroup analyses, such as to assess the impact of SCT or insurance type, or statistical testing were performed in this study. In addition, physician opinion on treatment selection was not captured in this study and therefore represents an avenue for further study.

In conclusion, this descriptive analysis indicates that community practices are increasingly adopting novel and effective therapies, as evidenced by the numeric rise in the utilization of triplet and quadruplet regimens, in addition to daratumumab-based treatments. However, there remains an opportunity to increase the use of these regimens in community practice to improve patient outcomes, as recommended in clinical guidelines and demonstrated in clinical trials. Future analysis leveraging the latest data will be essential to quantify the current and evolving uptake of quadruplet regimens as they become more widely used as 1L treatment in real-world clinical practice and the impact of these regimens on patient outcomes.

## Data Availability

The original contributions presented in the study are included in the article/**Supplementary Material**, Further inquiries can be directed to the corresponding author.
