# Supplementary material for: Front-line treatment approaches in multiple myeloma: real-world data from a community-based oncology network
Source: Front Oncol. 2026 Apr 22;16:1736892. doi: 10.3389/fonc.2026.1736892 (PMC13147281; doi:10.3389/fonc.2026.1736892)
Supplement: Supplementary file 1 [file DataSheet1.docx]

**Supplementary Material**

**Supplementary Figure 1.** Patients selected from the iKnowMed database to be included in the analysis.

Total number of patients included in the study (N = 3592)

Patients excluded (N = 6928):

- Received corticosteroids only or did not receive NCCN recommended MM drugs on or after MM diagnosis date (N = 4479)
- <18 years of age on index date or no 1L information available (N = 1678)
- Evidence of non-MM cancer (excluding plasmacytomas) during the baseline period (N = 91)
- Evidence of amyloid light-chain amyloidosis during the study period (N = 22)
- Evidence of clinical trial involvement any time on or prior to the index date (N = 6)
- Evidence of either autologous or allogeneic SCT any time on or prior to index date (N = 1)
- ≥1 year between MM diagnosis and start of 1L treatment (N = 161)
- Incomplete patient or regimen records (N = 490)

Patients newly diagnosed with MM between January 1, 2015 to December 31, 2022 based on ICD-10 C90.0x assessed for eligibility (N = 10,520)

1L, front-line. MM, multiple myeloma. NCCN, National Comprehensive Cancer Network. SCT, stem cell transplant.

**Supplementary Figure 2.** Distribution of VRd, VCyd, Vd, and daratumumab-based 1L treatment regimens by age group.

1L, front-line. Dara, daratumumab. VCyd, bortezomib-cyclophosphamide-dexamethasone. Vd, bortezomib-dexamethasone. VRd, bortezomib-lenalidomide-dexamethasone.
